# Supernormal Stimulus Begging Calls of Brood‐Parasitic Nestlings Depress the Parental Care in an Uncommon Host

**DOI:** 10.1002/ece3.71820

**Published:** 2025-07-20

**Authors:** Li Tian, Ruiying Han, Jingyi Su, Shuting Jia, Cuiping Yi, Jieru Wen, Zhengwang Zhang, Donglai Li, Yu Liu

**Affiliations:** ^1^ Life Science and Technology School Lingnan Normal University Zhanjiang China; ^2^ Key Laboratory for Biodiversity Sciences and Ecological Engineering, Ministry of Education, College of Life Sciences Beijing Normal University Beijing China; ^3^ State Key Laboratory of Environmental Criteria and Risk Assessment Chinese Research Academy of Environmental Sciences Beijing China; ^4^ Provincial Key Laboratory of Animal Resource and Epidemic Disease Prevention, College of Life Sciences Liaoning University Shenyang China

**Keywords:** begging, brood parasitism, feeding frequency, parental investment, playback

## Abstract

During the nestling period, brood‐parasitic birds stimulate host parents to provide food through complex visual and auditory signals, including emitting supernormal stimuli in the form of begging calls to increase the feeding frequency. However, whether the begging calls of brood‐parasitic nestlings act as a universal type of supernormal stimulus signal and their effects on less common host species still require further research. In this study, we used playback recordings to verify the impact of the begging calls of Common Cuckoo ( *Cuculus canorus* ) nestlings on the parental care behavior of host Barn Swallow ( *Hirundo rustica* ) parents. Contrary to our expectations, the results showed that male Barn Swallow parents decreased their feeding frequency in response to both types of Common Cuckoo nestling calls (cuckoo nestlings reared by the Oriental Reed Warbler *Acrocephalus orientalis* /the Barn Swallow), while females decreased their feeding frequency in response to the begging calls of Common Cuckoo nestlings reared by the common host (the Oriental Reed Warbler). Additionally, brood size, temperature, and weather all affected the feeding frequency in the Barn Swallow. This study supports the idea that the supernormal stimulus of brood‐parasitic nestling begging calls does not always work as a universal signal; the behavioral adaptations formed by parasitic birds in response to common hosts may lead to reduced fitness when utilizing uncommon hosts, for example, the Barn Swallow.

## Graphical abstract

The exaggerated begging calls of brood‐parasitic nestlings, for example, the Common Cuckoo, could serve as a supernormal stimulus to the host parents, but their effectiveness in uncommon host species remains unclear. In this study, we tested the effect of cuckoo nesting begging calls on an uncommon host species, the Barn Swallow, with manipulative playback experiments. Contrary to our expectations, we found that male Barn Swallows significantly reduced their feeding frequency in response to cuckoo nestling calls, suggesting that this signal inhibited parental investment in a less common host species.

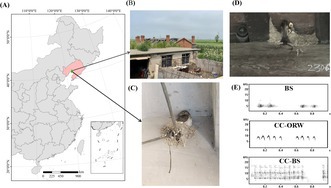

## 1 Introduction

Nestlings of altricial birds usually seek parental care using begging behavior, including making begging calls and giving begging gestures (Ottosson et al. 1997; Wright and Leonard 2007). While begging behavior is of direct benefit (i.e., food) to nestlings, they pay direct (energy consumption or predation risk) or indirect costs (sibling fitness reduction). Thus, begging is usually considered an honest signal (Cotton et al. 1996; Mock et al. 2011). However, for brood‐parasitic bird species, their nestlings pay little indirect costs for their begging behavior as they are not genetically related to host nestlings (if there is any) sharing the same nest (Boncoraglio et al. 2009; Redondo 1993). As a consequence, these nestlings stimulate their foster parents to gain more food resources using exaggerated begging calls, mainly including making louder or more intense calls, mimicking host (single nestling or whole brood) begging calls, emitting universal calls which can stimulate hosts widely, adjusting begging calls phenotypically, etc. (Soler 2017a; Stevens 2013). For instance, the begging calls of a Common Cuckoo ( *Cuculus canorus* ) nestling parasitizing an Eurasian Reed Warbler ( *Acrocephalus scirpaceus* ) nest stimulate foster parents equally to a whole brood of warbler nestlings (Davies et al. 1998), and they also stimulated higher feeding frequency in the host Red‐winged Blackbirds ( *Agelaius phoeniceus* , Li and Hauber 2021); thus begging call signals are considered supernormal stimuli to hosts. Brood‐parasitic nestlings exploit their hosts using these supernormal stimulus signals, gaining greater direct benefits and improving individual fitness consequently (Grim and Honza 2001; Redondo 1993).

Parental birds make provision for nestlings based on both their need (e.g., begging) and their condition. For instance, the feeding frequency of Great Tit ( *Parus major* ) parents is higher when making artificial playback of nestling begging calls, which simulates a situation that the nestling need is stronger (Bengtsson and Rydén 1983). Furthermore, parental provision is also limited by the foraging ability of parental birds: In the Barn Swallow ( *Hirundo rustica* ), parents feed more frequently to nestlings with higher need or in better condition; however, if nestling need goes beyond their foraging ability, parent Barn Swallows feed nestlings equally without discrimination (Saino et al. 2000). Previous research has shown that the supernormal stimulus signals given by brood‐parasitic nestlings can promote the feeding frequency of foster parents (Soler 2017a; Wright and Leonard 2007). For example, no matter for host (the House Wren *Troglodytes aedon* ) or non‐host (the Great Tit) species, when the begging calls of brood‐parasitic nestlings (the Shiny Cowbird *Molothrus bonariensis* , which shares the nest with host chicks) were played to parental birds, both species were stimulated to behave at a higher feeding frequency (Gloag and Kacelnik 2013). However, for nestmate‐evictor brood parasites, for example, the Common Cuckoo, which eventually eliminate their potential competitors such as other eggs or nestlings from the nest and are raised as the sole inhabitants of the foster parents' nest (Moskát et al. 2017), there lacks research testing whether their nestling begging calls are universal supernormal stimuli to uncommon hosts.

Though the Barn Swallow used to be considered a non‐host species for the Common Cuckoo (Davies and Brooke 1989), studies in recent years have shown that the Barn Swallow is a confirmed host for the Common Cuckoo; cuckoo nestlings could survive until fledging in the Barn Swallow nests under the care of parental Barn Swallows (Li et al. 2013; Liang et al. 2013; Liu et al. 2025; Su et al. 2017). Meanwhile, the Barn Swallow tries to avoid brood parasitism by attacking brood parasites directly, rejecting foreign eggs, and nesting in close proximity to human habitats (Liang et al. 2013; Yang et al. 2015; Yu et al. 2016; Li, Bai, et al. 2020). During brooding, except for the needs and conditions of nestlings, factors such as brood size, time of day, insect abundance, sexual selection, etc. also influence the feeding frequency of parental Barn Swallows (Boncoraglio et al. 2008; Møller 1994; A. Turner 2006). In this study, we aim to test whether the begging calls of Common Cuckoo nestlings are a supernormal stimulus to the Barn Swallow parents, that is, promoting their parental care investment. We predict that the begging calls of Common Cuckoo nestlings could act as a universal supernormal stimulus, and the feeding frequency of Barn Swallow parents will increase upon this stimulus.

## 2 Methods

### 2.1 Study Site and Species

**FIGURE 1:**
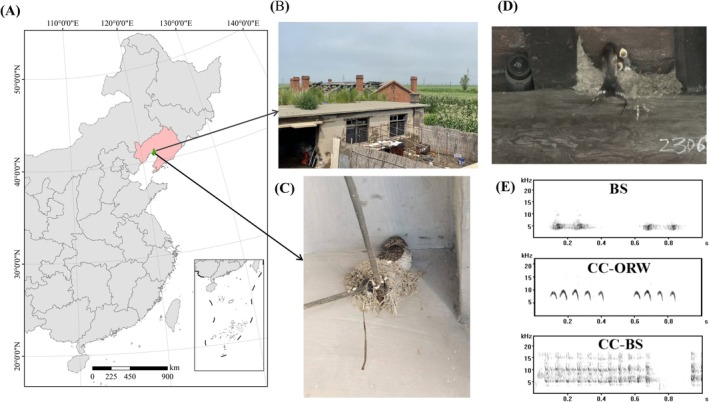
The location, habitat, and experimental setup of this study. We conducted this study in the Liao River Estuary National Nature Reserve, Panjin City, Liaoning Province, China (A), and the nesting locations and habitats of Barn Swallows are shown in (B). In 2022, a Common Cuckoo nestling parasitizing a Barn Swallow nest was found in a nearby village (C). A screenshot of the playback experiment video (D) and typical spectrograms of the begging calls used in the experiment are also shown (E, Top: Barn Swallow nestling begging calls, BS; Raw: The begging calls of a Common Cuckoo nestling from an Oriental Reed Warbler nest, CC‐ORW; Bottom: The begging calls of a Common Cuckoo nestling from a Barn Swallow nest, CC‐BS).

This study was conducted in the Liao River Estuary National Nature Reserve (40.9668° N–41.0243° N, 121.6729° E–121.7433° E), Panjin City, Liaoning Province of northeastern China from May to August in the years 2019, 2021, and 2023 (Figure 1). Panjin is a coastal city located near sea level, characterized by a warm temperate monsoon climate. The average annual temperature is 8.3°C, with annual precipitation averaging around 600 mm (Zhao et al. 2008). Within the reserve, Barn Swallows mainly nest in traditional brick and tile houses, in which workers used to live when harvesting reed, and the main habitat is reed pond (Li, Bai, et al. 2020). Common Cuckoos were commonly seen during the breeding season in the reserve, for which the main host is the Oriental Reed Warbler ( *Acrocephalus orientalis* , Li, Li, et al. 2020), and the local Common Cuckoo parasitism rate on the warbler was 16.6% (62 out of 373, Li et al. 2016). At the same time, although no brood parasitism events of the Common Cuckoo on Barn Swallows were found in an earlier study (0 out of 349, Li, Bai, et al. 2020), we observed one case of brood parasitism each year from 2021 to 2024 in a village around the nature reserve (Figure 1).

We searched for Barn Swallow nests from early May each year and monitored them once a nest was considered active. Typically, a female Barn Swallow lays 2–6 eggs per clutch in the study site, and the average clutch size is 4.62 ± 0.80 (mean ± SD) in the years 2019 and 2021 (Tian et al. 2023). We visited each nest daily after 12 days of incubation and recorded the hatching date of the first nestling. We captured adult Barn Swallows using mist nets set near the windows of the houses during the daytime or using sweep nets at night. Adult Barn Swallows were banded with a unique combination of coloured plastic and coloured metal rings on each of their legs, and sex was determined by the presence (females) or absence (males) of a brood patch (Liu et al. 2023).

### 2.2 Recording Begging Calls

We recorded the begging calls of nestlings using a Digital Audio Recorder (DR‐100MKIII, TASCAM, USA) with a sample rate of 44.1 kHz and sampling accuracy of 24 bits. Begging calls were recorded from natural nests in good weather conditions. While Barn Swallow nestlings fledge between 18 and 23 days of age, given the rapid growth and high resource demands of 11‐day‐old Barn Swallow nestlings, we chose individuals at this age as the experimental subjects (Li 2017). We tied the recorder to the bottom of Barn Swallow nests and recorded the natural begging calls of 11‐day‐old Barn Swallow nestlings for 1–1.5 h. Similarly, we recorded the natural begging calls of 12‐day‐old Common Cuckoo nestlings parasitizing Oriental Reed Warbler nests for 1 h per nest. In total, we recorded the begging calls of Barn Swallow nestlings from ten Barn Swallow nests and the begging calls of Common Cuckoo nestlings from ten Oriental Reed Warbler nests in the reserve, 2018. We clipped continuous sentences randomly selected from the pool of recorded Barn Swallow nestling audio and synthesized a piece of 5 min audio, and we clipped ten pieces of continuous 20‐s‐long sentences randomly selected from the pool of recorded Common Cuckoo audio and synthesized them into one piece for the playback experiment (Raven Pro 1.5.0, Cornell Lab of Ornithology, USA). In 2022, we also successfully recorded the begging calls of a Common Cuckoo nestling parasitizing a Barn Swallow nest (duratio*n* = 14.20 min, *n* = 1) with a cellphone (P40 Pro+, Huawei, China) from a village nearby the reserve. Based on its body metrics (body mass = 56.92 g, body length = 16.3 cm, wing length = 11.0 cm), the Common Cuckoo nestling was estimated to be around 20 days old. Although the recording quality of mobile phones may not be as good as that of a digital audio recorder, this was the best option we could take under the circumstances at that time.

### 2.3 Begging Call Playback Experiment

In this study, we tested whether the begging calls of Common Cuckoo nestlings are a supernormal stimulus to Barn Swallows with a playback experiment. We recorded the feeding behavior of parental Barn Swallows while playing one of three types of nestling's begging calls, which included the two types of Common Cuckoo nestling begging calls reared by the Barn Swallow or the Oriental Reed Warbler (experimental treatment, i.e., CC‐BS and CC‐ORW), Barn Swallow nestling begging calls (positive control treatment, i.e., BS), or playing no begging calls (blank control treatment, i.e., no playback). For each nest, the blank control treatment was given first, followed by the remaining treatments in random order, with a minimum interval of 10 min between each on the same day, and each treatment lasted for an hour. In each treatment, the audio synthesised as described above was played, and we aimed to finish the experiment of all treatments (three treatments in 2019 and 2021: blank, BS, CC‐ORW; four treatments in 2023: blank, BS, CC‐ORW, CC‐BS) when the nestlings were 12–13 days old. While we avoided conducting the experiment under severe weather, for each nest the experiment was finished within 3 days.

We used a portable speaker (S1, Zealot, Shenzhen, China), which was fastened within 30 cm around the Barn Swallow nest to play the nestling begging calls, while recording the feeding behavior of parental Barn Swallows using a camcorder (FDR‐AX30, Sony, Japan). The sound pressure of each playback treatment was measured at a distance of 2.5 m from the speaker, and the sound pressure level slightly differed between the three treatments (BS: 65.81 ± 0.76 dB; CC‐ORW: 62.28 ± 0.76 dB; CC‐BS: 59.49 ± 0.76 dB, all shown in mean ± SE. One‐way ANOVA: *F*_2,87_ = 17.26, *p* < 0.01). In the blank control group experiment, the portable speaker was also fixed in the same position but did not play any sound. Current weather and temperature were recorded at the start of the experiment, and we divided the weather and starting time of day into three categories for analysis (weather: sunny, cloudy, drizzle; time of day: morning 07:00–09:00, noon 09:00–12:00, afternoon 12:00–15:00). For each video, we played it using Potplayer (1.7.12248.0, Daum, Korea) and calculated the feeding frequency of male and female parental Barn Swallows (times per hour). In 2019, experiments were conducted on 17 nests for the blank control treatment, BS treatment, and CC‐ORW treatment. In 2021, experiments were conducted on 28 nests for each of these treatments. In 2023, experiments were conducted on 48 nests for the blank control treatment, 43 nests for the BS treatment, 45 nests for the CC‐ORW treatment, and 45 nests for the CC‐BS treatment.

### 2.4 Statistical Analyses

All analyses were conducted in R Language V4.5.0 (R Core Team 2025) and RStudio V2024.09.0 (RStudio Team 2024). Generalized linear mixed models (GLMMs) were constructed to analyze which factor could affect the feeding frequency of male or female Barn Swallows using the R package *lme4*. The playback treatment, nestling age, brood size, date, time of day, weather, year, playback order, and temperature were included as fixed factors, and nest ID was included as a random factor. Firstly, we tested for multicollinearity among the above variables using the variance inflation factor (VIF, function *ols_vif_tol* from R package *olsrr*). Factors were reserved for subsequent analyses if all VIFs were less than three (Zuur et al. 2010). Secondly, we chose the best models based on the corrected Akaike information criterion (AICc) among all candidate models, of which models with the lowest AICc score or ΔAICc < 2 were considered the best (function *dredge* and *subset* from R package *MuMIn*). Further, the parameters of the best models were conditionally averaged if there was more than one equally effective (ΔAICc < 2) model. In GLMMs, the response variables were male feeding frequency or female feeding frequency, and a Poisson distribution was selected to describe the error structures of the response variables. Additionally, to control for potential false positive results due to multiple comparisons in the GLMMs, we also performed pairwise comparisons using Tukey's HSD for the differences between designed playback treatments in the models with the lowest AICc value (function *emmeans* and *pairs* from R package *emmean*s).

Considering that CC‐BS treatment was only given in year 2023, we also constructed GLMM models with the data of year 2019/2021 and the data of year 2023, separately. In addition, we constructed GLMM models using the average feeding frequency of each nestling (parental feeding frequency/brood size) as the response variable to test whether the begging calls of Common Cuckoo nestlings would affect the quantity of food received by each Barn Swallow nestling. In results, means are given with standard errors (SE) and the significance level of all statistical tests is *α* = 0.05.

## 3 Results

**FIGURE 2:**
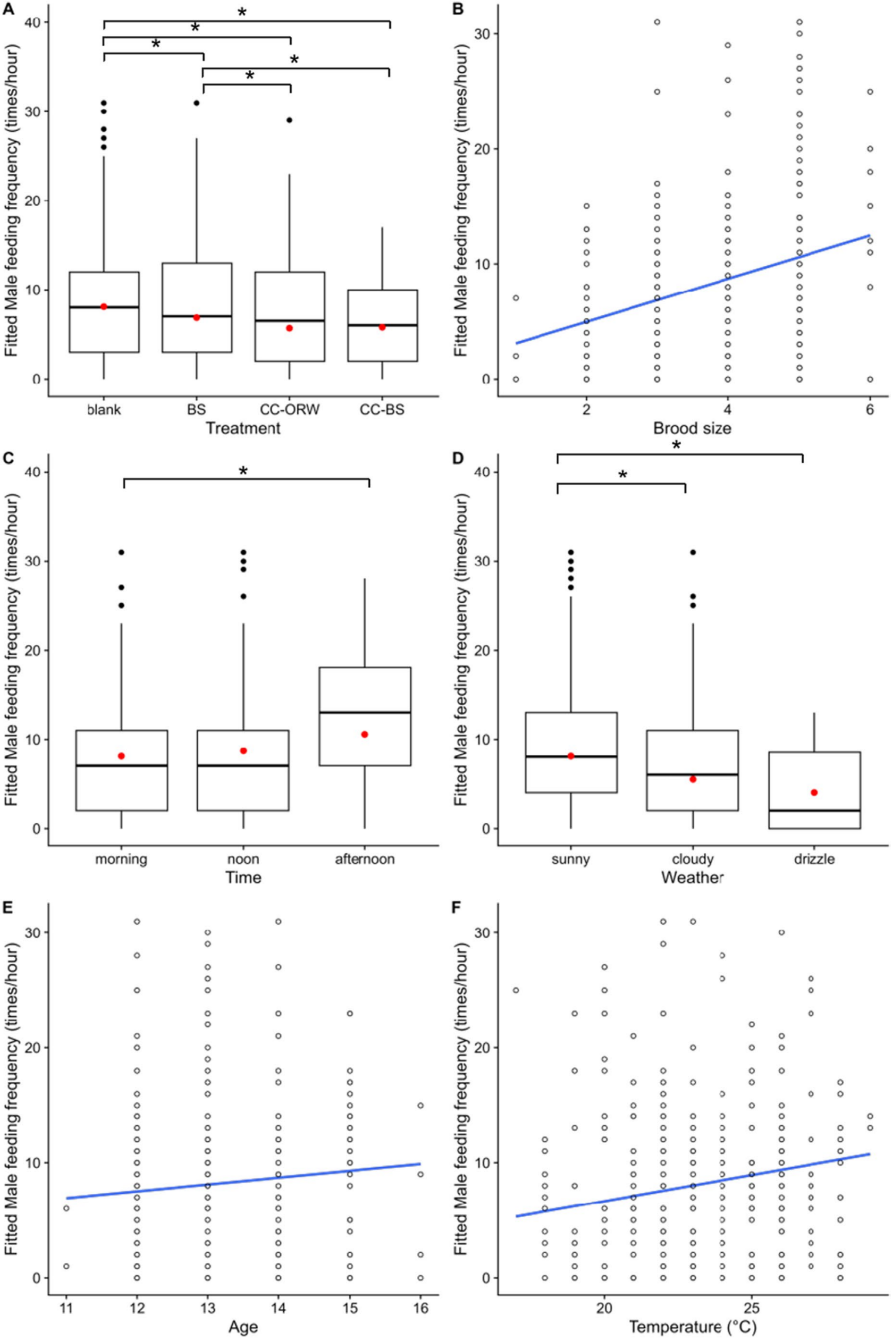
The feeding frequency of male Barn Swallow decreased upon the begging calls of Common Cuckoo nestlings (A), while increased with brood size (B). The feeding frequency also differed between different time of day (C) and different weather (D), and increased with nestling age (E) and temperature (F). The red and black circle dots in panels A, C, and D represent means and outliers, respectively. * indicates significant differences from GLMM results, and predicted values of the feeding frequency from the GLMM with the lowest AICc value and simple regression lines are shown.

The result of the GLMMs showed that the feeding frequency of male Barn Swallows in the CC‐ORW experimental treatments was significantly lower than that in the blank control treatment (CC‐ORW vs. blank, *p* < 0.001, Table 1) and the positive control treatment (CC‐ORW vs. BS, *p* < 0.001, Table 1). A similar pattern was also observed in the CC‐BS treatment (CC‐BS vs. blank, *p* < 0.001; CC‐BS vs. BS, *p* = 0.020; Table 1), while there was no significant difference in the feeding frequency between the CC‐ORW and CC‐BS experimental treatments (CC‐ORW vs. CC‐BS, *p* = 0.178, Table 1, Figure 2). Moreover, nestling age, brood size, temperature, and weather were all significantly correlated with the feeding frequency of male Barn Swallows.

**TABLE 1:** GLMMs testing the relationship between the feeding frequency of male Barn Swallows and the playback treatment, playback order, year, nestling age, brood size, temperature, date, time of day, and weather.

(A) Best models	AICc	ΔAICc	*w*_i_
Age + brood size + temperature + time + playback order + weather + year	2261.03	0.00	0.14
Age + brood size + date + temperature + time + playback order + weather	2261.45	0.42	0.11
Age + brood size + temperature + time + playback order + weather	2261.59	0.56	0.10
Age + brood size + date + temperature + time + playback order + weather + year	2262.50	1.47	0.06
Age + brood size + playback order, + temperature + time + playback order + weather + year	2262.88	1.85	0.05
Age + brood size + temperature + playback order + weather + year	2262.98	1.95	0.05

(B) Averaged parameters	Estimate ± SE	*p*	*Z*	Adjusted *p*
BS versus blank	0.16 ± 0.06	0.010	2.57	0.042
CC‐ORW versus blank	0.35 ± 0.06	< 0.001	5.35	< 0.001
CC‐BS versus blank	0.34 ± 0.08	< 0.001	4.14	< 0.001
CC‐ORW versus BS	0.19 ± 0.05	< 0.001	3.49	0.002
CC‐ORW versus CC‐BS	0.01 ± 0.08	0.178	0.86	0.995
CC‐BS versus BS	0.17 ± 0.07	0.020	2.33	0.100
Age	0.07 ± 0.03	0.030	2.18	
Brood size	0.27 ± 0.08	< 0.001	3.48	
Date	2.72 × 10^−3^ ± 3.54 × 10^−3^	0.559	0.58	
Temperature	0.06 ± 0.02	0.002	3.11	
Playback order	−0.02 ± 0.03	0.554	0.59	
Noon versus morning	−0.08 ± 0.06	0.209	1.26	0.456
Afternoon versus morning	−0.28 ± 0.11	0.011	2.56	0.034
Afternoon versus noon	−0.20 ± 0.11	0.056	1.92	0.143
Cloudy versus sunny	0.39 ± 0.07	< 0.001	5.65	< 0.001
Drizzle versus sunny	0.70 ± 0.17	< 0.001	4.10	< 0.001
Drizzle versus cloudy	0.32 ± 0.17	0.067	1.84	0.165

*Note:* (A) Best models selected based on AICc and the null model. (B) Averaged parameters (estimates ± SE and *p*‐values) of the best models. *w*_i_, AICc weight. Adjusted *p*‐values using Tukey's HSD test for the differences between designed playback treatments in the model with the lowest AICC value were also shown. BS, the begging calls of Barn Swallow nestlings; CC‐ORW, the begging calls of Common Cuckoo nestlings reared by the Oriental Reed Warbler; CC‐BS, the begging calls of a Common Cuckoo nestling reared by the Barn Swallow.

Abbreviations: BS, the begging calls of Barn Swallow nestlings; CC‐BS, the begging calls of a Common Cuckoo nestling reared by the Barn Swallow; CC‐ORW, the begging calls of Common Cuckoo nestlings reared by the Oriental Reed Warbler.

Though the blank control treatment (blank) and the positive control treatment (BS) showed significant difference when examining the data of 3 years (BS vs. blank, *p* = 0.010, Table 1), no significant difference were found when the data of year 2023 (*p* = 0.082, Table S1) or the data of year 2019 and 2021 were modelled separately (*p* = 0.279, Table S2). Furthermore, the average feeding frequency of each nestling in the CC‐ORW experimental treatments was significantly lower than that in the blank control treatment (CC‐ORW vs. blank, *p* = 0.041, Table S5). Additionally, weather conditions also showed an interannual effect on the feeding frequency of male parents (Tables S1 and S2).

**FIGURE 3:**
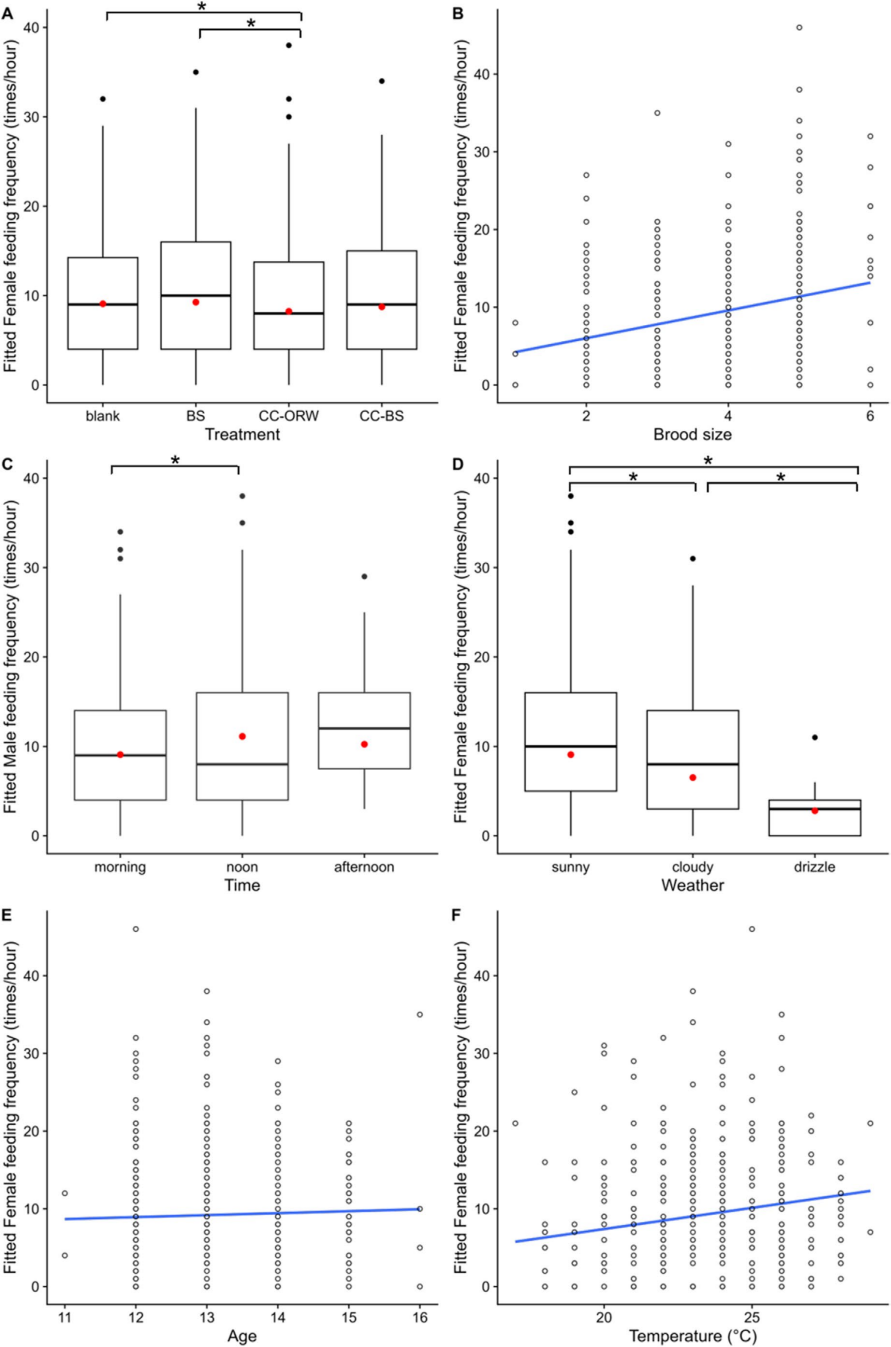
The feeding frequency of female Barn Swallow decreased upon the begging calls of Common Cuckoo nestlings (A), while increased with brood size (B). The feeding frequency also differed between different time of day (C) and different weather (D), and increased with nestling age (E) and temperature (F). The red and black circle dots in panels A, C, and D represent means and outliers, respectively. * indicates significant differences from GLMM results, and predicted values of the feeding frequency from the GLMM with the lowest AICc value and simple regression lines are shown.

We found a different pattern when analyzing the feeding frequency of the female Barn Swallows (Table 2, Figure 3). For female Barn Swallows, the feeding frequency in the CC‐ORW experimental treatments was also significantly lower than that in the blank control treatment (CC‐ORW vs. blank, *p* = 0.037) and the positive control treatment (CC‐ORW vs. BS, *p* = 0.013, Table 2), but there were no significant differences between other designed playback treatments (all *p* > 0.05, Table 2), and the average feeding frequency of each nestling was consistent across all treatments (the playback treatment was not included in the best models, Table S6). When only analyzing data from the years 2019/2021, the feeding frequency of female Barn Swallows also showed significant differences between CC‐ORW and blank treatments (CC‐ORW vs. blank, *p* = 0.032, Table S4), as well as between CC‐ORW and BS treatments (CC‐ORW vs. BS, *p* < 0.001, Table S4). Additionally, brood size, weather, and time of day (noon vs. morning) exhibited interannual variation in their effects on the feeding frequency of female parents (Tables S3 and S4).

**TABLE 2:** GLMMs testing the relationship between the feeding frequency of female Barn Swallows and the playback treatment, playback order, year, nestling age, brood size, temperature, date, time of day, and weather.

(A) Best models	AICc	ΔAICc	*w*_i_
Brood size + temperature + time + playback + weather	2263.82	0.00	0.13
Brood size + date + temperature + time + playback + weather	2264.35	0.53	0.10
Brood size + temperature + time + weather	2264.43	0.61	0.09
Age + brood size + temperature + time + playback + weather	2264.94	1.11	0.07
Brood size + date + temperature + time + weather	2265.28	1.45	0.06
Brood size + playback order + temperature + time + playback + weather	2265.52	1.70	0.05
Age + brood size + date + temperature + time + playback order+ weather	2265.79	1.97	0.05

(B) Averaged parameters	Estimate ± SE	*p*	*Z*	Adjusted *p*
BS versus blank	−0.01 ± 0.05	0.884	0.15	0.976
CC‐ORW versus blank	0.11 ± 0.05	0.037	2.09	0.178
CC‐BS versus blank	0.05 ± 0.06	0.460	0.74	0.922
CC‐ORW versus CC‐BS	0.06 ± 0.06	0.311	1.01	0.776
CC‐ORW versus BS	0.12 ± 0.05	0.013	2.49	0.063
CC‐BS versus BS	0.05 ± 0.06	0.373	0.89	0.776
Age	0.03 ± 0.03	0.034	0.96	
Brood size	0.23 ± 0.08	0.003	3.00	
Date	5.49 ± 4.56	0.230	1.20	
Temperature	0.06 ± 0.02	< 0.001	3.75	
Playback order	0.01 ± 0.02	0.495	0.68	
Noon versus morning	−0.20 ± 0.05	< 0.001	4.02	< 0.001
Afternoon versus morning	−0.12 ± 0.10	0.235	1.19	0.456
Afternoon versus noon	0.08 ± 0.10	0.405	0.83	0.682
Cloudy versus sunny	0.33 ± 0.06	< 0.001	5.59	< 0.001
Drizzle versus sunny	1.18 ± 0.20	< 0.001	5.84	< 0.001
Drizzle versus cloudy	0.85 ± 0.20	< 0.001	4.25	< 0.001

*Note:* (A) Best models selected based on AICc and the null model. (B) Averaged parameters (estimates ± SE and *p*‐values) of the best models. *w*_i_ = AICc weight. Adjusted *p*‐values using Tukey's HSD test for the differences between designed playback treatments in the model with the lowest AICc value were also shown.

## 4 Discussion

In this study, we tested whether the begging calls of Common Cuckoo nestlings were a supernormal stimulus to an uncommon host species, the Barn Swallow, using a manipulative playback experiment. Contrary to our prediction, neither the begging calls of Common Cuckoo nestlings raised by Barn Swallows nor by Oriental Reed Warblers did stimulate parent Barn Swallows to increase their feeding frequency. Especially, the feeding frequency of male Barn Swallows decreased upon Common Cuckoo nestling begging calls, while female Barn Swallow parents reduced their feeding frequency only in response to the begging calls of Common Cuckoo nestlings reared by common hosts. The inhibitory effect of the begging calls of Common Cuckoo nestlings reared by the uncommon host (Barn Swallow) showed a sexual difference between male and female Barn Swallows. Thus, diverging from previous studies which suggested that supernormal stimulus signals enhance parental provisioning, our results indicate that the begging calls of Common Cuckoo nestlings could reduce the provisioning frequency of a common host, especially that of males.

### 4.1 The Supernormal Stimuli of Nestmate‐Evictor Brood‐Parasitic Nestlings Are Not Universal

Brood parasites usually choose their hosts based on breeding strategies (brood reducer or clutch‐size adjuster) and relative body size (Soler 2017a). For instance, cuckoo nestlings kill nestmate host nestlings to monopolize the food provision of foster parents, while brood parasitic cowbirds always need to compete with nestmate host nestlings for food resources (Davies 2000; Soler 2017b). For the former, the food resource needed by Common Cuckoo nestlings is always equal to a whole brood of host nestlings due to their relatively larger body size, and Common Cuckoo nestlings evolved to mimic the begging calls of a whole host brood (Davies et al. 1998; Kilner et al. 1999). For the latter, under the assumption that foster parents prefer to feed larger nestlings, cowbird nestlings need to compete with host nestlings through begging behavior, especially when their body size is relatively smaller than that of host nestlings (Dearborn and Lichtenstein 2005). Theoretically, no matter whether there is a need to compete with nestmates, brood parasite nestlings benefit from making supernormal stimulus signals to promote the food provision of foster parents irrespective of energy consumption or predation risk. For example, when testing the effects of begging call playbacks of nestmate‐evictor Common Cuckoos or nestmate‐sharing Brown‐headed Cowbirds ( *Molothrus ater* ) upon parental Red‐winged Blackbird feeding patterns, the results showed that Common Cuckoo calls elicited more parental feeding trips and the amount of food delivered than cowbird calls (Li and Hauber 2021). In this study, the begging calls of Common Cuckoo nestlings did not work for Barn Swallows and even resulted in an inhibitory effect, illustrating that this type of supernormal stimulus was not a widely adapted universal signal, but was species‐specific. The phenomenon that this supernormal stimulus adapted to their main hosts (the Oriental Reed Warbler) was not adapted to an uncommon host (the Barn Swallow) supports that the cost is high to develop a universal supernormal stimulus signal for nestmate‐evictor brood parasites.

In this study, we tried our best to avoid pseudo‐replication: for the CC‐ORW and BS treatments, we played begging calls of Common Cuckoo or Barn Swallow nestlings from multiple different nests (CC‐ORW: *N* = 10; BS: *N* = 10), and the calls heard by returning adult Barn Swallows seemed largely random. However, due to the difficulty in obtaining begging calls of Common Cuckoos parasitizing Barn Swallows, we repeatedly used begging calls of a Common Cuckoo nestling from only one Barn Swallow nest. Another potential flaw in the design of this study is that, due to the scarcity of Common Cuckoos parasitizing Barn Swallows, although we immediately recorded the begging calls upon discovering the parasitism, the Common Cuckoo nestling whose calls were used in the experiment was still relatively older (20 days old, CC‐BS treatment). We hope that in future studies we will collect a larger and more diverse set of begging call recordings to further validate our findings and enhance their generalizability.

### 4.2 Sexual Differences in Feeding Frequency

Parental investment is the result of a balance between the efforts of both parents. Within breeding pairs, each sex may respond differently to the changing demands of parental care. These sexual differences might be related to the division of parental care between male and female parents (Porras‐Reyes et al. 2021). For example, a study conducted on the Pale‐breasted Thrush ( *Turdus leucomelas* ) in Spain found that males had a higher food provisioning rate and provided more food to larger broods, while females showed no response to brood size (Haddad et al. 2024). In this study, there was no significant difference in the feeding frequency between female and male Barn Swallows upon the blank control treatment (*t*‐test: T = −0.88, df = 183.42, *p* = 0.38). However, the begging calls of both Common Cuckoo nestlings decreased the feeding frequency of male Barn Swallows, while only the calls from Common Cuckoo nestlings raised by their common host, the Oriental Reed Warbler, affected female feeding frequency. This may be partly because parasitic Common Cuckoo nestlings can flexibly develop their begging calls to tune into the communication system or provisioning strategies (Madden and Davies 2006). Due to males having larger Passerine song‐control regions (SCRs) than females (Nottebohm and Arnold 1976), which are crucial for song learning, production, and perception (Nealen and Perkel 2000), male Barn Swallows may be more sensitive to the begging calls of nestlings, thereby recognizing and reducing the feeding frequency upon Common Cuckoo nestling begging calls.

On the other hand, the different responses of male and female parents to the begging calls of Common Cuckoos might also be related to the sex‐dependent strategies of parental investment. Previous research has shown that male and female parents can show compensatory parenting behavior: they respond more to each other's behavior rather than to the calls of the nestlings, for example, the feeding rate of one parent can influence the brooding behavior of its partner independently of the nestlings' behavior (Hinde and Kilner 2007). Thus, it is possible that when male Barn Swallows reduce their feeding frequency in response to the begging calls of Common Cuckoo nestlings, females may compensate for the reduced feeding by the males. This could lead to a stable (or even higher) feeding frequency of females; for instance, females did not show a significant decrease in their feeding frequency upon the CC‐BS treatment.

### 4.3 Other Factors Affecting Feeding Frequency

This study shows that the feeding frequency of Barn Swallow parents increased with the number of nestlings. On the other hand, the study supports that the feeding frequency of the Barn Swallow parents largely depends on the abundance of food resources. Under good weather conditions, the feeding frequency of parental birds was significantly higher than during unsatisfactory weather, which is consistent with previous research results (Bryant and Turner 1982; A. K. Turner 1980). The feeding frequency of Barn Swallows varies throughout the day, and the patterns of feeding frequency changes are inconsistent across different populations (A. Turner 2006; Zieliński and Wojciechowski 1999). In this study, the feeding frequency of Barn Swallows was higher in the afternoon and midday than in the morning. The possible explanation for this result is that the activity and quantity of their food, mainly insects, increase gradually with rising temperatures, causing the feeding frequency of Barn Swallow parents to fluctuate accordingly (van Dijk et al. 2024).

## 5 Conclusions

In this study, we conducted a manipulative playback experiment on Barn Swallows during the nestling period, using recordings of Common Cuckoo nestling begging calls parasitizing Barn Swallow and Oriental Reed Warbler nests, respectively. By comparing the feeding frequency while playing Common Cuckoo nestling begging calls, Barn Swallow nestling begging calls, and a blank control, we found that the Common Cuckoo nestling begging calls, regardless of whether they came from the same or different host nests, reduced the feeding frequency of Barn Swallows, especially the feeding frequency of male Barn Swallows. Therefore, we concluded that in this parasite–host system, the exaggerated stimulus signals from Common Cuckoo nestlings inhibited rather than stimulated the parental investment of a less common host species.

## Author Contributions

**Li Tian:** writing – original draft (equal), writing – review and editing (equal). **Ruiying Han:** investigation (equal). **Jingyi Su:** investigation (equal). **Shuting Jia:** investigation (equal), writing – original draft (equal). **Cuiping Yi:** investigation (equal). **Jieru Wen:** investigation (equal). **Zhengwang Zhang:** project administration (equal), resources (equal), supervision (equal). **Donglai Li:** conceptualization (equal). **Yu Liu:** conceptualization (equal), investigation (equal), writing – original draft (equal), writing – review and editing (equal).

## Ethics Statement

All the methods were carried out according to the Regulations for the Administration of Affairs Concerning Experimental Animals (Ministry of Science and Technology, China, revised in March 2017). The study was approved by the Ethical and Animal Welfare Committee (Approval No.: CLS‐EAW‐2021‐018).

## Consent

The authors have nothing to report.

## Conflicts of Interest

The authors declare no conflicts of interest.

## Supporting information


**Table S1.** GLMMs testing the relationship between the feeding frequency of male Barn Swallows and the playback treatment, nestling age, brood size, temperature, date, time of day and weather in year 2023. (A) Best models selected based on AICc and the null model. (B) Averaged parameters (estimates ± SE and *p*‐values) of the best models. *w*_i_ = AICc weight.

## Data Availability

Data and associated data products are held by the Chinese Research Academy of Environmental Sciences and may be available through a public records request. All code for analyses are located on Dryad: https://datadryad.org/stash/dataset/doi:10.5061/dryad.8931zcs1f.
