# Supplementary material for: Supernormal Stimulus Begging Calls of Brood‐Parasitic Nestlings Depress the Parental Care in an Uncommon Host
Source: Ecol Evol. 2025 Jul 20;15(7):e71820. doi: 10.1002/ece3.71820 (PMC12276819; doi:10.1002/ece3.71820)
Supplement: Supplementary file 1 — Table S1. GLMMs testing the relationship between the feeding frequency of male Barn Swallows and the playback treatment, nestling age, brood size, temperature, date, time of day and weather in year 2023. (A) Best models selected based on AICc and the null model. (B) Averaged parameters (estimates ± SE and p‐values) of the best models. w i = AICc weight. [file ECE3-15-e71820-s001.docx]

**Table S1. GLMMs testing the relationship between the feeding frequency of male Barn Swallows and the playback treatment, nestling age, brood size, temperature, date, time of day and weather in year 2023.** (A) Best models selected based on AICc and the null model. (B) Averaged parameters (estimates ± SE and P-values) of the best models. *w*_i_ = AICc weight.

| (A) Best models | | AICc | | ΔAICc | | | *w*_i_ |  |
| --- | --- | --- | --- | --- | --- | --- | --- | --- |
| age + brood size + temperature + playback order+ weather | | 1219.72 | | 0.00 | | | 0.13 |  |
| brood size + temperature + playback order+ weather | | 1219.75 | | 0.03 | | | 0.12 |  |
| brood size + playback order + temperature + playback + weather | | 1220.22 | | 0.50 | | | 0.10 |  |
| brood size + date + temperature + playback order+ weather | | 1221.18 | | 1.47 | | | 0.06 |  |
| age + brood size + date + temperature + playback order+ weather | | 1221.53 | | 1.81 | | | 0.05 |  |
|  |  | |  | |  |  | | |
| (B) Averaged parameters | Estimate ± SE | | P | | Z value | Adjusted P | | |
| BS vs. blank | -0.14 ± 0.08 | | 0.082 | | 1.74 | 0.218 | | |
| CC-ORW vs.blank | -0.25 ± 0.09 | | 0.004 | | 2.87 | 0.008 | | |
| CC-BS vs.blank | -0.32 ± 0.09 | | <0.001 | | 3.62 | 0.001 | | |
| CC-ORW vs. BS | -0.11 ± 0.08 | | 0.204 | | 1.27 | 0.519 | | |
| CC-ORW vs. CC-BS | 0.07 ± 0.08 | | 0.420 | | 0.81 | 0.875 | | |
| CC-BS vs. BS | -0.17 ± 0.08 | | 0.037 | | 2.08 | 0.143 | | |
| age | 0.07 ± 0.05 | | 0.141 | | 1.47 |  | | |
| brood size | 0.20 ± 0.09 | | 0.031 | | 2.16 |  | | |
| date | 0.01 ± 0.01 | | 0.432 | | 0.79 |  | | |
| temperature | 0.06 ± 0.02 | | 0.011 | | 2.54 |  | | |
| playback order | 0.04 ± 0.03 | | 0.182 | | 1.33 |  | | |
| cloudy vs. sunny | 0.09 ± 0.10 | | 0.361 | | 0.91 | 0.608 | | |
| drizzle vs. sunny | -0.58 ± 0.20 | | 0.003 | | 2.93 | 0.007 | | |
| drizzle vs. cloudy | -0.67 ± 0.20 | | <0.001 | | 3.33 | 0.002 | | |

**Table S2. GLMMs testing the relationship between the feeding frequency of male Barn Swallows and the playback treatment, nestling age, brood size, temperature, year, date, time of day and weather in year 2019 and 2021.** (A) Best models selected based on AICc and the null model. (B) Averaged parameters (estimates ± SE and P-values) of the best models. *w*_i_ = AICc weight.

| (A) Best models | | | | AICc | | ΔAICc | | *w*_i_ |  |
| --- | --- | --- | --- | --- | --- | --- | --- | --- | --- |
| brood size + temperature + time + playback + weather + year | | | | 1004.98 | | 0.00 | | 0.21 |  |
| age + brood size + temperature + time + playback + weather + year | | | | 1005.75 | | 0.76 | | 0.15 |  |
| brood size + temperature + playback + weather + year | | | | 1006.96 | | 1.98 | | 0.08 |  |
|  | |  | | | |  | |  |  |
| (B) Averaged parameters | Estimate ± SE | | P | | Z value | | Adjusted P | | |
| BS vs. blank | -0.10 ± 0.09 | | 0.279 | | 1.08 | | 0.587 | | |
| CC-ORW vs. blank | -0.36 ± 0.09 | | <0.001 | | 3.82 | | <0.001 | | |
| CC-ORW vs. BS | -0.26 ± 0.07 | | <0.001 | | 3.58 | | 0.001 | | |
| age | 0.06 ± 0.05 | | 0.197 | | 1.29 | |  | | |
| brood size | 0.36 ± 0.11 | | <0.001 | | 3.32 | |  | | |
| year2021 | 0.50 ± 0.23 | | 0.028 | | 2.20 | |  | | |
| temperature | 0.07 ± 0.02 | | 0.003 | | 2.97 | |  | | |
| noon vs. morning | 0.06 ± 0.09 | | 0.501 | | 0.67 | | 0.843 | | |
| afternoon vs. morning | 0.32 ± 0.12 | | 0.010 | | 2.58 | | 0.026 | | |
| afternoon vs. noon | 0.26 ± 0.13 | | 0.043 | | 2.02 | | 0.081 | | |
| cloudy vs. sunny | -0.83 ± 0.10 | | <0.001 | | 8.39 | | <0.001 | | |
| drizzle vs. sunny | -0.51 ± 0.36 | | 0.159 | | 1.41 | | 0.301 | | |
| drizzle vs. cloudy | 0.32 ± 0.36 | | 0.377 | | 0.88 | | 0.635 | | |

**Table S3. GLMMs testing the relationship between the feeding frequency of female Barn Swallows and the playback treatment, nestling age, brood size, temperature, date, time of day and weather in year 2023.** (A) Best models selected based on AICc and the null model. (B) Averaged parameters (estimates ± SE and P-values) of the best models. wi = AICc weight.

| (A) Best models | | | | AICc | | ΔAICc | | *w*_i_ | |
| --- | --- | --- | --- | --- | --- | --- | --- | --- | --- |
| age + brood size + playback order + temperature + weather | | | | 1238.08 | | 0.00 | | 0.19 | |
| brood size + temperature + weather | | | | 1239.04 | | 0.96 | | 0.12 | |
| age + brood size + temperature + weather | | | | 1239.62 | | 1.54 | | 0.09 | |
|  | |  | | | |  | |  |  |
| (B) Averaged parameters | Estimate ± SE | | P | | Z value | | Adjusted P | | |
| brood size | 0.27 ± 0.09 | | 0.004 | | 2.92 | |  | | |
| temperature | 0.09 ± 0.02 | | <0.001 | | 4.33 | |  | | |
| playback order | 0.07 ± 0.03 | | 0.541 | | 1.93 | |  | | |
| age | -0.10 ± 0.06 | | 0.104 | | 1.63 | |  | | |
| cloudy vs. sunny | 0.04 ± 0.09 | | 0.685 | | 0.41 | | 0.841 | | |
| rainy vs. sunny | -1.29 ± 0.27 | | <0.001 | | 4.82 | | <0.001 | | |
| rainy vs. cloudy | -1.32 ± 0.26 | | <0.001 | | 5.08 | | <0.001 | | |

**Table S4. GLMMs testing the relationship between the feeding frequency of female Barn Swallows and the playback treatment, nestling age, brood size, temperature, year, date, time of day and weather in year 2019 and 2021.** (A) Best models selected based on AICc and the null model. (B) Averaged parameters (estimates ± SE and P-values) of the best models. wi = AICc weight.

| (A) Best models | | AICc | | ΔAICc | | *w*_i_ | |  |
| --- | --- | --- | --- | --- | --- | --- | --- | --- |
| temperature + time + playback + weather | | 987.34 | | 0.00 | | 0.17 | |  |
| brood size + temperature + time + playback + weather | | 987.49 | | 0.15 | | 0.16 | |  |
| brood size + date + temperature + time + playback + weather | | 988.55 | | 1.21 | | 0.09 | |  |
| brood size + temperature + time + playback + weather | | 988.64 | | 1.30 | | 0.09 | |  |
| temperature + time + playback + weather + year | | 988.97 | | 1.63 | | 0.08 | |  |
|  | |  | |  | |  | |  |
| (B) Averaged parameters | Estimate ± SE | | P | | Z value | | Adjusted P | |
| BS vs. blank | 0.12 ± 0.07 | | 0.063 | | 1.86 | | 0.587 | |
| CC-ORW vs. blank | -0.15 ± 0.07 | | 0.032 | | 2.15 | | 0.079 | |
| CC-ORW vs. BS | -0.30 ± 0.07 | | <0.001 | | 4.32 | | <0.001 | |
| brood size | 0.17 ± 0.11 | | 0.113 | | 1.59 | |  | |
| date | 6.89×10^-3^ ± 5.97×10^-3^ | | 0.252 | | 1.14 | |  | |
| temperature | 0.06 ± 0.02 | | 0.007 | | 2.69 | |  | |
| year2021 | 0.21 ± 0.21 | | 0.329 | | 0.98 | |  | |
| noon vs. morning | 0.31 ± 0.08 | | <0.001 | | 3.96 | | <0.001 | |
| afternoon vs. morning | 0.15 ± 0.12 | | 0.215 | | 1.24 | | 0.408 | |
| afternoon vs. noon | -0.15 ± 0.12 | | 0.209 | | 1.26 | | 0.328 | |
| cloudy vs. sunny | -0.66 ± 0.08 | | <0.001 | | 7.70 | | <0.001 | |
| drizzle vs. sunny | -0.60 ± 0.31 | | 0.059 | | 1.89 | | 0.141 | |
| drizzle vs. cloudy | 0.06 ± 0.32 | | 0.843 | | 0.20 | | 0.975 | |

**Table S5. GLMMs testing the relationship between the average male feeding frequency of each nestling and the playback treatment, playback order, age, brood size, year, date, time, weather and temperature in year 2019, 2021 and 2023.** (A) Best models selected based on AICc and the null model. (B) Averaged parameters (estimates ± SE and P-values) of the best models. wi = AICc weight.

| (A) Best models | | | AICc | | ΔAICc | | *w*_i_ | |
| --- | --- | --- | --- | --- | --- | --- | --- | --- |
| date + time + playback + weather | | | 1147.97 | | 0.00 | | 0.05 | |
| date + playback + weather | | | 1148.69 | | 0.72 | | 0.03 | |
| date + time + weather | | | 1148.73 | | 0.76 | | 0.03 | |
| temperature + playback + weather | | | 1148.79 | | 0.82 | | 0.03 | |
| date + weather | | | 1149.03 | | 1.06 | | 0.03 | |
| temperature + playback + weather + year | | | 1149.10 | | 1.13 | | 0.03 | |
| temperature + weather + year | | | 1149.17 | | 1.20 | | 0.03 | |
| date + temperature+ playback + weather | | | 1149.36 | | 1.39 | | 0.02 | |
| date + playback order + time + playback + weather | | | 1149.44 | | 1.47 | | 0.02 | |
| age + date + time + playback + weather | | | 1149.75 | | 1.78 | | 0.02 | |
| date + temperature + time + playback + weather | | | 1149.76 | | 1.79 | | 0.02 | |
| temperature + weather | | | 1149.76 | | 1.79 | | 0.02 | |
| date + playback order + playback + weather | | | 1149.79 | | 1.82 | | 0.02 | |
|  | |  | | |  | |  |  |
| (B) Averaged parameters | Estimate ± SE | | P | Z value | | Adjusted P | | |
| age | 0.03 ± 0.05 | | 0.539 | 0.61 | |  | | |
| date | 7.59×10^-3^ ± 3.70×10^-3^ | | 0.041 | 2.04 | |  | | |
| year2021 | 0.17 ± 0.19 | | 0.390 | 0.86 | |  | | |
| year2023 | -0.15 ± 0.19 | | 0.410 | 0.82 | |  | | |
| playback order | 0.04 ± 0.04 | | 0.362 | 0.91 | |  | | |
| temperature | 0.04 ± 0.03 | | 0.120 | 1.56 | |  | | |
| noon vs. morning | 0.05 ± 0.10 | | 0.620 | 0.50 | | 0.813 | | |
| afternoon vs. morning | 0.39 ± 0.18 | | 0.028 | 2.19 | | 0.057 | | |
| afternoon vs. noon | 0.35 ± 0.18 | | 0.058 | 1.90 | | 0.133 | | |
| BS vs. blank | -0.02 ± 0.11 | | 0.829 | 0.22 | | 0.997 | | |
| CC-ORW vs. blank | -0.23 ± 0.11 | | 0.041 | 2.04 | | 0.133 | | |
| CC-BS vs. blank | -0.25 ± 0.14 | | 0.085 | 1.73 | | 0.343 | | |
| CC-ORW vs. BS | -0.21 ± 0.11 | | 0.052 | 1.94 | | 0.190 | | |
| CC-ORW vs. CC-BS | 0.02 ± 0.14 | | 0.906 | 0.12 | | 1.000 | | |
| CCBS vs.BS | -0.22 ± 0.14 | | 0.114 | 1.58 | | 0.429 | | |
| cloudy vs. sunny | -0.30 ± 0.11 | | 0.007 | 2.68 | | 0.013 | | |
| drizzle vs. sunny | -0.82 ± 0.32 | | 0.012 | 2.51 | | 0.021 | | |
| drizzle vs. cloudy | -0.52 ± 0.32 | | 0.111 | 1.59 | | 0.208 | | |

**Table S6. GLMMs testing the relationship between the average female feeding frequency of each nestling and the playback treatment, playback order, age, brood size, year, date, time, weather and temperature in year 2019, 2021 and 2023.** (A) Best models selected based on AICc and the null model. (B) Averaged parameters (estimates ± SE and P-values) of the best models. wi = AICc weight.

| (A) Best models | | | AICc | | ΔAICc | | *w*_i_ | |
| --- | --- | --- | --- | --- | --- | --- | --- | --- |
| temperature + weather | | | 1228.34 | | 0.00 | | 0.09 | |
| date + time + weather | | | 1228.34 | | 0.00 | | 0.09 | |
| date + temperature + time + weather | | | 1228.52 | | 0.19 | | 0.08 | |
| temperature + time + weather | | | 1228.62 | | 0.28 | | 0.07 | |
| date + temperature + weather | | | 1229.59 | | 1.25 | | 0.05 | |
| playback order + temperature + weather | | | 1230.22 | | 1.88 | | 0.03 | |
|  | |  | | |  | |  |  |
| (B) Averaged parameters | Estimate ± SE | | P | Z value | | Adjusted P | | |
| date | 6.80×10^-3^ ± 4.39×10^-3^ | | 0.123 | 1.54 | |  | | |
| temperature | 0.05 ± 0.02 | | 0.034 | 2.12 | |  | | |
| playback order | 0.01 ± 0.03 | | 0.655 | 0.45 | |  | | |
| noon vs. morning | 0.21 ± 0.09 | | 0.026 | 2.23 | | 0.013 | | |
| afternoon vs. morning | 0.23 ± 0.19 | | 0.219 | 1.23 | | 0.191 | | |
| afternoon vs. noon | 0.02 ± 0.18 | | 0.897 | 0.13 | | 0.939 | | |
| cloudy vs. sunny | -0.24 ± 0.10 | | 0.020 | 2.33 | | 0.070 | | |
| drizzle vs. sunny | -1.23 ± 0.37 | | <0.001 | 3.35 | | 0.003 | | |
| drizzle vs. cloudy | -0.99 ± 0.36 | | 0.006 | 2.74 | | 0.021 | | |
